# The patient reported intraocular lens questionnaire (PR-ILQ): content validity, psychometric performance, and use in a regulated clinical trial to evaluate safety and effectiveness outcomes

**DOI:** 10.1186/s41687-025-00968-0

**Published:** 2025-12-12

**Authors:** Alan L. Shields, Nina Galipeau, Leighann Litcher-Kelly, Alejandro Moreno-Koehler, Jimmy Chacko

**Affiliations:** 1 Adelphi Values, One Lincoln Street, Suite 2900, Boston, MA 02111 USA; 2 Lenstec, Inc, 1765 Commerce Ave. N., St. Petersberg, Fl 33716 UK

**Keywords:** Patient-reported outcomes (PROs), Intraocular lens (iol) replacement, Patient reported intraocular lens questionnaire (pR-ILQ), Content validity, Psychometric performance, Cataracts

## Abstract

**Background:**

Patient-reported outcomes (PROs) are underutilized in medical device evaluations, including in ophthalmology and intraocular lens (IOL) replacements. This article summarizes research conducted to develop the Patient-Reported Intraocular Lens Questionnaire (PR-ILQ) and support its content validity and psychometric performance. Use of the PR-ILQ in a regulated clinical trial to support new product approval decisions, secondary effectiveness claims, and safety observations is also discussed.

**Results:**

The PR-ILQ, developed with ophthalmology experts (*n* = 10) and use of qualitative data generated from patients during concept elicitation (*n* = 44) and cognitive debriefing (*n* = 32) interviews, includes the Vision Correction Scale (VCS, three items), Vision Disturbance Scale (VDS, eight items), and IOL Replacement Satisfaction Scale (IOL-RSS, five items). Each assessment is distinct, and items within each are scored independently to address treatment benefit hypotheses. With its content validity established, the PR-ILQ was administered to 271 subjects (mean age = 68.3 years) participating in a clinical trial to evaluate the safety and effectiveness of an asymmetric segmented multifocal IOL (SBL-INI-02–13). Descriptively, item scores behaved as expected, with clustering of VCS and VDS scores at more- and less-severe levels at study entry and exit, respectively, though scores were distributed across response options across timepoints. Test-retest reliability results mostly indicate “fair”/“good” (weighted Kappa [KW]/intraclass correlation coefficient [ICC] > 0.40 to 0.60) to “excellent” (KW/ICC > 0.75) reproducibility for VCS, VDS, and IOL-RSS items in two independent analyses. Construct validity hypotheses, including those associated with sensitivity to change, were supported via correlational analysis showing a pattern of expected relationships among PR-ILQ items and with other variables including ratings of visual acuity. For example, reduced use of vision correction at near (*r* = −0.28), intermediate (*r* = −0.42), and far (*r* = −0.57) distances were more strongly related to overall lens satisfaction at end of study than improved visual acuity at those same distances (*r* = −0.25. −0.26, and −0,14).

**Conclusions:**

The PR-ILQ is content valid and early evidence suggests it is capable of producing reliable scores upon which valid conclusions may be drawn when administered among patients undergoing cataract lens replacement surgery. Acknowledging limitations and need for additional psychometric evaluation, the assessment recently supported a new product approval decision, secondary effectiveness claims, and safety observations. Together, this indicates that the PR-ILQ, along with the evidentiary basis and suggestions for use presented herein, will be of immediate value to IOL replacement outcomes researchers, regulators, and other stakeholders interested in generating evidence to inform health care decisions and improving cataracts patients’ lives.

**Trial registration:**

Clinicaltrials.gov, NCT02487160. Registered 25 June 2015, https://clinicaltrials.gov/study/NCT02487160.

## Background

In medical device development, benefit is evaluated by survival and how patients function and feel [1]. Survival benefits are not often targeted in ophthalmology; therefore, vision-related treatments are evaluated based on how a patient functions and feels. Clinical outcome assessments (COAs), direct measures of how patients function or feel, are therefore critical for determining treatment benefits in ophthalmology trials. Most frequently used COAs include performance outcomes (e.g., how well a subject correctly calls out letters on an eye chart) and patient-reported outcomes (PROs; e.g., a patient’s rating of blurry vision on a 0 to 10 scale where 0 = not blurry and 10 = extremely blurry) [2, 3].

Cataracts afflict most humans, cause the eye lens to cloud, and lead to blurry vision and other vision disturbances that hinder everyday activities requiring sight [4]. Their treatment involves replacing the blurry lens with an artificial intraocular lens (IOL) [5], of which there are many approved by the United States (US) Food and Drug Administration (FDA). Historically, the indication for use (IFU) statements for these products [6] have focused on “visual correction” and for patients “who desire near, intermediate, and distance vision with increased spectacle independence” [7](p.1).

As regulatory agencies become more patient-centric [8–11], the IFU language for IOL products is evolving to focus on evidence of improved vision function and realistic expectations based on visual performance and patient self-reports. For example, defined as clarity or sharpness of vision [12], visual acuity (VA) is often determined by an individual’s ability to see and correctly call out letters on the Early Treatment Diabetic Retinopathy Study (ETDRS) chart, which transforms performance into quantitative estimates of VA called LogMAR values presented at near, intermediate, and far distances [13]. Results from these assessments support treatment effectiveness claims such as: relative to the comparator, IOL device X provides improved near and intermediate visual acuity, while maintaining comparable VA at far distances.

Though improved VA is the gold standard outcome for IOL replacement [14], patients [15, 16], healthcare providers [17, 18], payers [19], and regulators [1, 20] encourage use of supplemental outcomes important to patients undergoing the treatment. Indeed, more recently approved IOL products include PRO-based information in IFU statements and supporting regulatory documents based on patient-reported post-treatment use of vision correction, visual disturbances, and treatment satisfaction [21]. Despite advances, PROs were included in only about half of FDA-evaluated medical devices from 2014 through 2020, and only about a third supported approval decisions (i.e., as primary or secondary endpoints) [22]. While these proportions were higher in the cohort including IOL replacements, results suggest a lack of PRO questionnaires with the necessary evidentiary basis for use in ophthalmology and IOL-replacement clinical studies to support new product review. This echoed the FDA and American Academy of Ophthalmology’s earlier conclusion that PRO questionnaires used in clinical trials for premium IOLs may lack proper development, psychometric evaluation, and relevance to patients or specific to clinical research [23].

The demand for PRO questionnaires in ophthalmic research and practice escalates [24], yet a gap still exists in acceptable PRO questionnaires for use in those contexts, including IOL replacement studies. In response, outcomes researchers are developing new PRO assessments for use in a variety of ophthalmic settings [25], including for cataract research [26], and it is within this context that the Patient-Reported Intraocular Lens Questionnaire (PR-ILQ) was created. We expect the assessment to be a useful tool for researchers, regulators, and other stakeholders interested in IOL replacement treatment outcomes.

**Fig. 1:**
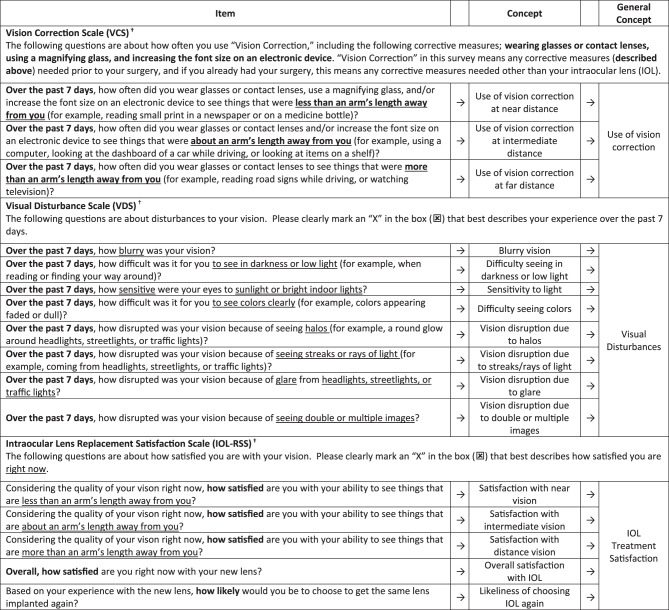
Conceptual Framework for the Patient-Reported Intraocular Lens Questionnaire^*^ ^*^Use of the PR-ILQ is prohibited without the express written permission of Lenstec, Inc ^†^Subjects in the clinical trial were given additional instruction asking them to answer all of the questions and to select only one answer for each question, and stating that there are no “right” or “wrong” answers to any of the questions and how to change answers if needed

Explicitly intended for use in IOL replacement clinical research, the PR-ILQ assesses use of vision correction to see at near, intermediate, and far distances (Vision Correction Scale [VCS]), visual disturbances (Vision Disturbance Scale [VDS]), and satisfaction with the implanted lens (IOL Replacement Satisfaction Scale [IOL-RSS]). The VCS, VDS, and IOL-RSS are each conceptually distinct, assess independently recognizable concepts, and are not intended to create a total or composite score (i.e., they are not intended to be combined conceptually, empirically, or interpretatively). In other words, as depicted the PR-ILQ’s conceptual framework (Fig. 1), the VCS, VDS, and IOL-RSS can each be considered as a distinct questionnaire under the umbrella of the PR-ILQ.

The objective of this report is to describe the development of the PR-ILQ as well as the research and evidence supporting its content validity and psychometric performance. These results are discussed in the context of an IOL device development program in which the PR-ILQ was used to assess treatment benefit and safety outcomes [21, 27].

## Methods

**Fig. 2:**
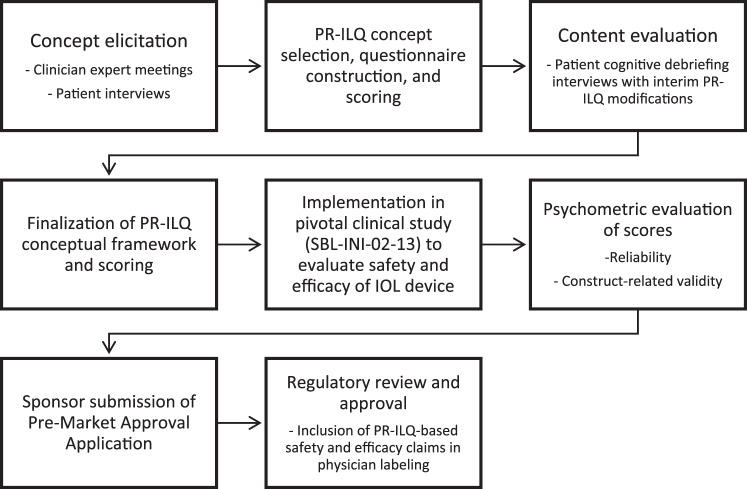
Patient-Reported Intraocular Lens Questionnaire Development and Use

Due to the lack of suitable PRO questionnaires for IOL clinical trials, research activities were initiated to create the PR-ILQ in ways consistent with regulatory guidance [28, 29] and best practices for measurement development [30, 31]. These activities are described below and summarized in Fig. 2.

### Concept elicitation

Concept elicitation (CE) reflects activities aimed at (1) understanding disease-related experiences relevant to a condition and important to patients who live with that condition and (2) justifying the inclusion of those experiences in PRO questionnaires [30]. CE was conducted with therapeutic area experts and patients.

*Clinical expert advice meeting*: Concepts relevant to cataract patients (pre- and post-IOL implantation) were identified first via a video-recorded 90-minute focus group with consented clinical experts from the US and United Kingdom (UK) (*N* = 10). Led by a trained moderator, the session was semi-structured to allow for an open discussion, and experts were asked to describe the symptoms of cataracts pre-IOL implantation and then to describe spectacle dependence, visual disturbances, and patient satisfaction following IOL implantation, based on their experience with different lens designs (i.e., monofocal, multifocal, and segmented bifocal IOLs). The recording was transcribed for analysis.

*Patient interviews*: Fourty-four one-on-one CE interviews with patients (*n* = 10 with current cataracts and *n* = 34 post-IOL implantation) were conducted across five clinical sites in the UK and US. Ethics approval was obtained from Aston University Ethics Committee in November 2013 and from Copernicus Group Independent Review Board® (CGIRB) in February 2014. To participate, patients provided written informed consent and satisfied all study inclusion and exclusion criteria, which were developed to match the intended target patient population (i.e., patients, as determined by the clinician, in good general and ocular health, with age-related cataracts requiring IOL implantation, and who could tolerate IOL surgery). Recruitment targets were primarily aimed at ensuring a diverse sample of patients who had either a current cataract condition or monofocal, multifocal, or segmented bifocal IOL replacement, in addition to demographic targets intended to be reflective of the broader patient population.

Most interviews (*n* = 42) were conducted in person, and two by telephone. Trained interviewers used a semi-structured guide to elicit the patient experience with IOLs and/or cataracts and then asked targeted probes to collect specific information (e.g., frequency, duration, and severity of a given experience like blurry vision). Interviews were audio-recorded, transcribed, anonymized, and analyzed using ATLAS.ti (ATLAS.ti Scientific Software Development GmbH; Berlin, Germany). Data analysis focused on (1) demonstrating saturation (as a justification of sample size, saturation reflects the point in data collection when little or no new relevant patient experience data emerges [32]), (2) determining the number of participants mentioning a particular concept as a relevant cataract or IOL experience, and (3) analyzing concepts within each IOL subgroup (i.e., monofocal, multifocal, and segmented bifocal participants). Cataract data were analyzed separately from IOL data.

### Concept selection, questionnaire construction, and scoring

Results from the expert focus group and patient CE interviews informed the selection of concepts for the PR-ILQ. After selecting measurement concepts, the questionnaire’s context of use and measurement parameters (e.g., recall period, response options, and format) were defined and the questionnaire was constructed. Consistent with treatment benefit hypothesis, scoring rules were developed.

### Content evaluation

The draft PR-ILQ was evaluated for readability, meaning, and comprehensiveness via cognitive debriefing (CD) interviews among patients with current cataracts (*n* = 3) or IOLs (*n* = 29) [31]. Approval to execute the CD interviews was received from CGIRB in February 2014, and study amendments were approved in September and November 2014. Patients were recruited from two US clinical sites, meeting inclusion/exclusion criteria and recruitment targets as in the CE interviews.

Interviews were conducted with consent, in person, audio-recorded, and lasted approximately 60 minutes, with questionnaire modifications tested iteratively. During the interview, participants completed the PR-ILQ using a “think-aloud” method to identify any unclear words, terms, or concepts [31]. Then the participant was asked to explain what each instruction, item, and response option meant to them and reported on any missing or repetitive concepts or items, the appropriateness of the examples and response options provided for each item, and any rewording suggestions.

### Psychometric evaluation

The PR-ILQ was included in an IRB-approved, prospective, subject-masked, randomized, two-arm, parallel-group study (SBL-INI-02–13) among consented adults with bilateral cataracts who underwent IOL replacement (clinicaltrials.gov identifier number: NCT02487160). In SBL-INI-02–13, the PR-ILQ was administered via paper assessment twice prior to the IOL replacement surgery (once each at Baseline and the pre-operative visit, Visit 00 and Visit 0A, respectively) and three times post-operatively at Visits 3A (30–60 days post-operative), 4A (120–180 days post-operative), and 5A (330–420 days post-operative). It is important to note that IOL-RSS Items 4 and 5 are specific to a newly implanted lens and, therefore, were only asked post-operatively (i.e., at Visits, 3A, 4A, and 5A). Conceptualized as three distinct questionnaires, the VCS, VDS, and IOL-RSS item scores were evaluated descriptively and in terms of reliability and construct-related validity on blinded data and using SAS® version 9.3 for Windows (SAS Institute, Cary, NC).

All analyses presented herein were pre-specified prior to conduct of analysis. Justification for the sample size was based on power calculations related to the primary efficacy analysis (not presented here). There is a cited need for guidance and a lack of consensus regarding a priori justifications for psychometric focused analysis sample size [33]; however, outcomes researchers have suggested *n* = 200 as sufficient for the descriptive, reliability, and validity analysis performed herein, though larger samples may be needed when considering more sophisticated data analytic choices [34, 35]. When data were missing, regardless of reason, no imputation methods were applied in the current psychometric analysis (i.e., they were labeled as missing). Additionally, as a first analysis aimed at understanding the psychometric performance of assessments administered in the exact context for which they were designed, no formal normality testing was conducted and no adjustments for multiplicity were made.

## Results

### Concept elicitation

The concepts most frequently reported by patients are presented in Table 1 alongside conclusions from the expert advice meeting. Together, these data served as a framework for the construction of the questionnaire.

**Table 1:** Patient reported cataract and intraocular lens-related concepts

Concept	Patient-reported Experience (*N* = 44)		Reported as most bothersome to patient (*N* = 44)		Endorsed by experts (*N* = 10)	
	Cataract^*;†^ (*n* = 40)	IOL^‡;†^ (*n* = 34)	Cataract^*;†^ (*n* = 40)	IOL^‡;†^ (*n* = 34)	Cataract	IOL
**Vision correction**						
Wearing glasses (or other corrective measures) for near vision^§^	37 (92.5%)	13 (38.2%)	12 (30.0%)	6 (17.6%)	YES	YES
Wearing glasses (or other corrective measures) for intermediate vision^**^	26 (65.0%)	8 (23.5%)	5 (12.5%)	2 (5.9%)	YES	YES
Wearing glasses (or using other corrective measures) for distance vision^††^	32 (80.0%)	2 (5.9%)	9 (22.5%)	0 (0%)	YES	YES
**Visual disturbances**						
Blurry vision^‡‡^	26 (65.1%)	17 (50.0%)	5 (12.5%)	8 (23.5%)	YES	YES
Sensitivity to light^‡‡^	14 (35.0%)	20 (58.8%)	6 (13.6%)	7 (20.6%)	NO	NO
Colors being less bright or more bright^‡‡;§§^	10 (25.0%)	17 (50.0%)	0 (0.0%)	0 (0.0%)	YES	YES
Difficulty seeing in darkness or low light^‡‡^	20 (50.0%)	8 (23.5%)	4 (10.0%)	0 (0%)	YES	YES
Seeing halos^‡‡^	9 (22.5%)	16 (47.1%)	2 (5.0%)	4 (11.8%)	YES	YES
Glare^‡‡^	7 (17.5%)	12 (35.3%)	1 (2.5%)	3 (8.8%)	YES	YES
Seeing rays or streaks of light^‡‡^	3 (7.5%)	8 (23.5%)	0 (0.0%)	3 (8.8%)	NO	YES
Double vision^‡‡^	4 (10.0%)	6 (17.6%)	2 (5.0%)	1 (2.9%)	NO	NO
Factors associated with IOL satisfaction and dissatisfaction						
Improved vision quality^***^	N/A	20 (58.8%)	N/A		YES	

Note: IOL: intraocular lens; N: sample size; N/A: not applicable.

^*^Patients who currently have or previously had cataracts and reported the concept as part of their cataract experience. Of note, *n* = 4 patients who had segmented bifocal IOL replacement with no prior diagnosis of cataracts were allowed in the study due to the lack of available patients with this lens type. These patients reported on their IOL experience only.

^‡^Patients who had undergone IOL implantation and reported the concept as part of their IOL experience. ^†^Frequency counts include spontaneous reports and those in response to a probed inquiry. ^§^Patients most frequently mentioned wearing glasses (*n* = 36/37 with cataract, *n* = 12/13 with IOL), contact lenses (*n* = 3/37 with cataract, *n* = 0/13 with IOL), or other corrective measures (e.g., use of magnifying glass or adjusting digital font size) (*n* = 8/37 with cataract, *n* = 3/13 with IOL). Counts are not mutually exclusive. ^**^Patients most frequently mentioned wearing glasses (*n* = 24/26), contact lenses (*n* = 2/26), or other corrective measures (e.g., adjusting distance from screen) (*n* = 1/26). Counts are not mutually exclusive. ^††^Patients most frequently mentioned wearing glasses (*n* = 31/32 with cataract, *n* = 2/2 with IOL) or contact lenses (*n* = 3/32 with cataract, *n* = 0/2 with IOL). Counts are not mutually exclusive. ^‡‡^Concept was reported as a visual disturbance by at least one-third of patients with segmented bifocal IOL. ^§§^Colors being less bright was reported as a cataract-related concept, colors being more bright was reported as an IOL-related concept. ^***^Though most patients (*n* = 28, 58.8%) indicated generally that improved vision quality would be related to their IOL replacement treatment, others were more specific, indicating that resolution of near (*n* = 12, 35.3%), intermediate (*n* = 11, 32.4%), and far (*n* = 8, 23.5%) were important to them as well (*n* values are not mutually exclusive)

*Clinical expert advice meeting*: Nine surgeons and one optometrist with IOL implant experience and/or treating patients with IOLs participated in the meeting. Most experts (*n* = 7) practiced in the US and three, in the UK. All of the experts who surgically implant IOLs and for whom there are descriptive data (*n* = 7) had been doing so for over 10 years and four, for 20 years or more. The number of IOLs the surgeons reported implanting per month ranged from 30 to 250, with total surgeries ranging from 5,500 to over 120,000.

During the focus group, experts affirmed that poor vision at any distance was relevant to patients with cataracts and that improved vision would reflect an important outcome for both the healthcare provider and patient. Experts also described the frustrating and life-limiting over-dependence that many patients have on use of glasses, contacts, and other vision correction choices (e.g., using a magnifying glass or increasing the font size on an electronic device). In this context, experts also affirmed that reduced dependence on use of vision correction choices would benefit patients and reflect improved VA. Interestingly, two experts mentioned that some patients are not bothered by using glasses after monofocal IOL implantation due to low expectations of vision restoration. However, advancements in IOL technology have led to higher expectations for improved vision and less reliance on vision correction.

Experts identified several visual disturbances associated with cataracts (i.e., disturbances that would reflect treatment benefit if reduced in the context of an effective IOL replacement) and the IOL replacement itself (i.e., disturbances that would reflect treatment benefit if avoided or minimized post-IOL replacement), including blurry vision, difficulty seeing in darkness or low light, colors being less bright or more bright, seeing halos, glare, floaters, and double vision (Table 1).

*Patient interviews*: Participants (*N* = 44) had a mean age of 65.3 years (standard deviation [SD] = 9.0), and the majority were female (*n* = 29, 65.9%) and white (*n* = 37, 84.1%). Among patients reporting education (*n* = 40), most completed at least some college or a certificate program (*n* = 17, 42.5%). Ten patients each (22.7%) had no IOL (i.e., the patient had a current cataract condition), monofocal lenses, or multifocal lenses, while 14 (31.8%) had bifocal segmented lenses.

Cataracts: A total of 29 cataract-related concepts were elicited spontaneously among patients with cataracts (*n* = 40), with 28 elicited in the first 30 interviews. Saturation was supported as little new data emerged during the final quartile of interviews. The most frequently reported were: reliance on vision correction for near vision (*n* = 37); difficulty seeing at near distance during activities such as reading, sewing, and writing (*n* = 36); reliance on vision correction for distance vision (*n* = 32); and difficulty seeing at far distance (*n* = 29), such as while driving/reading road signs, watching television, and recognizing people’s faces.

IOL-related: A total of 44 IOL-related concepts were elicited spontaneously among patients with IOLs (*n* = 34), with 40 elicited in the first 26 interviews. Saturation was supported, as little new data emerged during the final quartile of interviews. The most frequently reported concepts were sensitivity to light (*n* = 20), difficulty seeing at near distance (*n* = 18), colors appearing brighter (*n* = 17), and blurry vision (*n* = 17). In addition, IOL patients reported on their level of satisfaction or dissatisfaction with their IOLs. The two most frequently reported concepts associated with IOL satisfaction or dissatisfaction were vision quality (*n* = 31) and independence from use of vision correct choices (*n* = 28).

### Concept selection, questionnaire construction, and scoring

The CE results and context-of-use variables were considered while creating the PR-ILQ instructions, items, recall periods, and response scales. Concept selection was based primarily on the frequency of concepts reported spontaneously from the CE samples along with concept relevancy to the segmented-bifocal IOL subgroup (*n* = 14) and bothersomeness to participants (Table 1). The reporting of the concept during the clinical expert advice meeting was also considered as supportive evidence to the patient reports (note: the number of experts endorsing each concept was not considered, as it might be misleading. For example, verbal endorsement of each concept’s relevancy was not explored with every expert during the meeting).

For implementation into clinical studies, the VCS, VDS, and IOL-RSS are presented as a single questionnaire; however, all are conceptually distinct, have their own scoring conventions, and are not intended to create a total or composite score (i.e., they are not intended to be combined empirically or interpretatively). In this context, as depicted the PR-ILQ’s conceptual framework (Fig. 1), because the VCS, VDS, and IOL-RSS each measure independently recognizable general concepts and bring an organizational structure to the broader assessment, they may be considered as three distinct questionnaires under the umbrella of the PR-ILQ.

- *Vision Correction Scale (VCS)*: The VCS has three items to assess use of vision correction (such as eyeglasses, contact lenses, and/or magnifying glass) at near, intermediate, and far distances over the past seven days. Given lack of diurnal variation in the concept of measurement, the seven-day recall period was justified to reduce assessment burden (e.g., as compared to a momentary or daily assessment) while not being so long as to introduce memory distortion or recall bias [36]. To capture frequency-related aspects of measurement (i.e., VCS items ask respondents “how often” they used vision correction choices, see Fig. 1), items are endorsed on a five-point verbal rating scale (VRS) anchored at 1 = Never and 5 = All of the time. VCS item scores range from 1 to 5, with higher scores indicating more frequent use of vision correction. Like measures of VA that independently focus on near, intermediate, and distance vision, items on the VCS are scored independently to test specific treatment hypotheses (e.g., one might hypothesize effect on both VA and reduced reliance on vision correction choices at near and intermediate distances and a non-inferior effect on VA and reduced reliance on vision correction choices at far distances relative to a control).
- *Vision Disturbance Scale (VDS)*: The VDS has eight items to assess visual disturbances (e.g., blurry vision) in the past seven days. Consistent with the VCS, the seven-day recall period was defensible for use in order to be long enough to capture experiences that may be less frequent (e.g., glare) while again, not being so long as to introduce memory distortion or recall bias [36, 37]. Because the VDS assesses severity of experience (see Fig. 1), the items are scored on an 11-point numeric rating scale (NRS) where 0 = no experience with the visual disturbance (e.g., not at all blurry) and 10 = severe experience (e.g., extremely blurry). VDS item scores range from 0 to 10, with higher scores indicating greater severity. Items are scored independently to assess treatment effect on specific disturbances.
- *IOL Replacement Satisfaction Scale (IOL-RSS)*: The IOL-RSS assesses patient satisfaction with IOL replacement treatment across five items based on their experience “right now,” in order to capture patients’ level of satisfaction at time of assessment [36, 37]. The first three items use a five-point VRS to assess satisfaction with their near, intermediate, and far vision quality in the present moment (1 = Extremely dissatisfied to 5 = Extremely satisfied). The latter two items use a five-point VRS to assess overall satisfaction with the new lens (1 = Extremely dissatisfied to 5 = Extremely satisfied) and how likely the respondent would be to choose the same lens (1 = Extremely unlikely to 5 = Extremely likely). IOL-RSS item scores range from 1 to 5, with higher scores indicating greater satisfaction. Items are scored independently to represent specific aspects of patient satisfaction. Note: Because IOL-RSS Items 4 and 5 assess satisfaction with new lens, they are not intended for pre-operative administration.

### Content evaluation

The mean age of participants in the CD interviews (*n* = 32) was 67.8 years (SD = 8.2), and 21 (65.6%) were female. Most participants reported their highest level of education as either having completed at least some college or a certificate program (*n* = 13, 40.6%) or having received a high school diploma or less (*n* = 12, 37.5%). According to clinicians, 16 participants (50.0%) had multifocal IOLs, 13 (40.6%) had monofocal IOLs, and 3 (9.4%) had current cataracts (i.e., no surgery or IOLs).

During Round 1 of CD interviews (*n* = 22), the draft PR-ILQ was debriefed in its entirety, and the VCS was subsequently revised to improve wording and presentation of response scale; two items in the VDS were modified to improve patient understanding, and it was determined that no modifications were needed to the original IOL-RSS. The modified VCS and VDS scales and the IOL-RSS were debriefed with an additional 10 participants in the final round of CD interviews. Two modified VCS versions were debriefed and well understood by participants; ultimately one version was selected for the final PR-ILQ based on patient-reported preference. Additionally, both the modified VDS and original IOL-RSS were well understood and relevant to and comprehensive of the patient experience; therefore, no additional modifications were made.

### Psychometric performance

Adults (*N* = 271) participated in a clinical study (SBL-INI-02–13) across 18 sites in the US to evaluate the safety and efficacy of a novel IOL replacement (SBL3 IOL). Participants had a mean age of 68.3 years (SD = 7.3) and were mostly female (*n* = 187, 69.0%) and Caucasian (*n* = 261, 96.3%).

*Descriptive statistics*: There was very little missing data, and responses for each of the VCS (Table 2), VDS (Table 3), and IOL-RSS (Table 4) items were distributed across all responses across timepoints, indicating that respondents utilized the range of response options available.

**Table 2:** Vision Correction Scale (VCS) item scores and reliability across timepoints

Item	Timepoint				
	Visit 00 (*n* = 175)	Visit 0A (*n* = 271)	Visit 3A (*n* = 221)	Visit 4A (*n* = 185)	Visit 5A (*n* = 121)
**Item 1 (near distance) (range = 1 to 5)**					
Mean (SD)	4.3 (1.2)	4.3 (1.2)	2.0 (1.5)	2.1 (1.5)	2.2 (1.6)
Median	5.0	5.0	1.0	1.0	1.0
1: Never used vision correction	8 (4.6%)	15 (5.5%)	131 (59.3%)	101 (54.6%)	67 (55.4%)
2: Used vision correction some of the time	16 (9.1%)	22 (8.1%)	35 (15.8%)	32 (17.3%)	17 (14.0%)
3: Used vision correction half of the time	6 (3.4%)	13 (4.8%)	8 (3.6%)	10 (5.4%)	5 (4.1%)
4: Used vision correction most of the time	28 (16.0%)	29 (10.7%)	19 (8.6%)	13 (7.0%)	7 (5.8%)
5: Used vision correction all the time	117 (66.9%)	192 (70.8%)	28 (12.7%)	29 (15.7%)	25 (20.7%)
Test-retest reliability (KW [95% CI])^*^	–	0.705 (0.571, 0.838)	0.939 (0.901, 0.976)	–	–
**Item 2 (intermediate distance) (range = 1 to 5)**					
Mean (SD)	4.2 (1.3)	4.1 (1.4)	1.6 (1.2)	1.8 (1.3)	1.8 (1.4)
Median	5.0	5.0	1.0	1.0	1.0
1: Never used vision correction	11 (6.3%)	32 (11.8%)	163 (73.8%)	124 (67.0%)	79 (65.3%)
2: Used vision correction some of the time	19 (10.9%)	23 (8.5%)	25 (11.3%)	26 (14.1%)	16 (13.2%)
3: Used vision correction half of the time	10 (5.7%)	10 (3.7%)	9 (4.1%)	7 (3.8%)	6 (5.0%)
4: Used vision correction most of the time	26 (14.9%)	32 (11.8%)	11 (5.0%)	11 (5.9%)	6 (5.0%)
5: Used vision correction all the time	109 (62.3%)	174 (64.2%)	13 (5.9%)	17 (9.2%)	14 (11.6%)
Test-retest reliability (KW [95% CI])^*^	–	0.652 (0.535, 0.769)	0.895 (0.827, 0.963)	–	–
**Item 3 (far distance) (range = 1 to 5)**					
Mean (SD)	3.9 (1.6)	4.0 (1.5)	1.2 (0.7)	1.4 (0.9)	1.5 (1.2)
Median	5.0	5.0	1.0	1.0	1.0
1: Never used vision correction	30 (17.1%)	44 (16.2%)	199 (90.0%)	149 (80.5%)	98 (81.0%)
2: Used vision correction some of the time	16 (9.1%)	15 (5.5%)	12 (5.4%)	21 (11.4%)	7 (5.8%)
3: Used vision correction half of the time	8 (4.6%)	12 (4.4%)	2 (0.9%)	2 (1.1%)	3 (2.5%)
4: Used vision correction most of the time	15 (8.6%)	22 (8.1%)	4 (1.8%)	7 (3.8%)	5 (4.1%)
5: Used vision correction all the time	106 (60.6%)	178 (65.7%)	4 (1.8%)	6 (3.2%)	8 (6.6%)
Test-retest reliability (KW [95% CI])^*^	–	0.811 (0.727, 0.895)	0.813 (0.655, 0.971)	–	–

Note: CI = confidence interval; IOL: intraocular lens; KW: weighted Kappa coefficient; n: sample size; SD: standard deviation. ^*^Test-retest reliability was assessed using KW and its 95% CI applied to data gathered (1) preoperatively between Visits 0A and 00 (administrations were, on average, 15.0 days apart [SD = 13.1, range = 0–88 days], *n* = 175) and (2) within Visit 3A (administrations were approximately 120 minutes apart, *n* = 209)

**Table 3:** Visual Disturbances Scale (VDS) item scores and reliability across timepoints

Item	Timepoint					
	Visit 00 (*n* = 175)	Visit 0A (*n* = 270)	Visit 3A (*n* = 221)		Visit 4A (*n* = 185)^*^	Visit 5A (*n* = 121)
**Item 1 (blurry vision) (range = 0 to 10)**						
Mean (SD)	6.5 (2.8)	6.0 (2.8)	3.1 (2.8)		2.7 (2.7)	2.6 (2.8)
Median	7.0	6.0	3.0		2.0	2.0
Min–Max	0.0–10.0	0.0–10.0	0.0–10.0		0.0–10.0	0.0–10.0
Test-retest reliability: ICC (95% CI)^†^	–	0.567 (0.458, 0.660)	0.902 (0.874, 0.925)		–	–
**Item 2 (difficult in darkness or low light) (range = 0 to 10)**						
Mean (SD)	6.3 (3.0)	5.9 (3.1)	2.1 (2.8)		2.0 (2.6)	1.9 (2.7)
Median	7.0	7.0	1.0		1.0	1.0
Min–Max	0.0–10.0	0.0–10.0	0.0–10.0		0.0–10.0	0.0–10.0
Test-retest reliability: ICC (95% CI)^†^	–	0.604 (0.501, 0.690)	0.833 (0.786, 0.870)		–	–
**Item 3 (sensitive to sunlight or bright light) (range = 0 to 10)**						
Mean (SD)	6.3 (3.2)	5.9 (3.3)	2.1 (2.8)		3.7 (3.3)	3.3 (3.2)
Median	7.0	7.0	1.0		3.0	2.0
Min–Max	0.0–10.0	0.0–10.0	0.0–10.0		0.0–10.0	0.0–10.0
Test-retest reliability: ICC (95% CI)^†^	–	0.620 (0.520, 0.703)	0.928 (0.907, 0.945)		–	–
**Item 4 (see colors clearly) (range = 0 to 10)**						
Mean (SD)	4.3 (3.1)	3.6 (3.2)	0.70 (1.6)		0.60 (1.5)	0.50 (1.3)
Median	5.0	3.0	0.0		0.0	0.0
Min–Max	0.0–10.0	0.0–10.0	0.0–9.0		0.0–10.0	0.0–6.0
Test-retest reliability: ICC (95% CI)^†^	–	0.515 (0.398, 0.616)	0.882 (0.848, 0.909)		–	–
**Item 5 (seeing halos) (range = 0 to 10)**						
Mean (SD)	5.6 (3.5)	5.4 (3.8)	3.3 (3.3)	2.6 (3.1)		2.2 (2.7)
Median	6.0	6.0	2.0	2.0		1.0
Min–Max	0.0–10.0	0.0–10.0	0.0–10.0	0.0–10.0		0.0–10.0
Test-retest reliability [ICC (95% CI)]^†^	–	0.770 (0.702, 0.824)	0.916 (0.891, 0.935)	–		–
**Item 6 (seeing streaks or rays of light) (range = 0 to 10)**						
Mean (SD)	5.3 (3.4)	5.0 (3.7)	2.9 (3.2)	2.4 (3.1)		2.2 (2.8)
Median	6.0	5.0	2.0	1.0		1.0
Min–Max	0.0–10.0	0.0–10.0	0.0–10.0	0.0–10.0		0.0–10.0
Test-retest reliability: ICC (95% CI)^†^	–	0.692 (0.607, 0.762)	0.906 (0.878, 0.927)	–		–
**Item 7 (glare) (range = 0 to 10)**						
Mean (SD)	6.4 (3.3)	6.1 (3.5)	3.1 (3.3)	2.8 (3.2)		2.5 (2.9)
Median	7.0	7.0	2.0	2.0		1.0
Min–Max	0.0–10.0	0.0–10.0	0.0–10.0	0.0–10.0		0.0–10.0
Test-retest reliability: ICC (95% CI)^†^	–	0.765 (0.695, 0.820)	0.925 (0.902, 0.942)	–		–
**Item 8 (seeing double or multiple images) (range = 0 to 10)**						
Mean (SD)	3.2 (3.3)	2.9 (3.4)	1.8 (2.9)	1.5 (2.7)		1.3 (2.5)
Median	2.0	1.0	0.0	0.0		0.0
Min–Max	0.0–10.0	0.0–10.0	0.0–10.0	0.0–10.0		0.0–10.0
Test-retest reliability: ICC (95% CI)^†^	–	0.598 (0.494, 0.685)	0.879 (0.844, 0.906)	–		–

Note: CI: confidence interval; ICC: intraclass correlation coefficient; Max: maximum value; Min: minimum value; n: sample size; SD: standard deviation. ^*^One subject did not complete VDS Item 1 at Visit 4A (*n* = 184). ^†^Test-retest reliability was assessed using ICC, 3A1 for absolute agreement in a two-way mixed-effects model (in which patients are a random effect, and time a fixed effect) and its 95% CI [38]. This analytic choice was applied to data gathered (1) preoperatively between Visits 0A and 00 (administrations were, on average, 15.0 days apart [SD = 13.1, range = 0–88 days], *n* = 175) and (2) within Visit 3A (administrations were approximately 120 minutes apart, *n* = 209)

**Table 4:** Intraocular lens Replacement Satisfaction Scale (IOL-RSS) item scores and reliability across timepoints

Item	Timepoint					
	Visit 00 (*n* = 170)	Visit 0A (*n* = 182)	Visit 3A (*n* = 220)	Visit 4A (*n* = 185)	Visit 5A (*n* = 121)	
**Item 1 (satisfaction with near vision) (range = 1 to 5)**						
Mean (SD)	2.1 (1.0)	2.1 (1.0)	3.8 (1.3)	3.9 (1.3)		3.9 (1.3)
Median	2.0	2.0	4.0	4.0		4.0
1: Extremely dissatisfied	47 (26.9%)	56 (20.7%)	15 (6.8%)	12 (6.5%)		6 (5.0%)
2: Dissatisfied	83 (47.4%)	74 (27.3%)	30 (13.6%)	25 (13.5%)		18 (14.9%)
3: Neither satisfied nor dissatisfied	16 (9.1%)	27 (10.0%)	25 (11.3%)	18 (9.7%)		10 (8.3%)
4: Satisfied	21 (12.0%)	25 (9.2%)	61 (27.6%)	49 (26.5%)		30 (24.8%)
5: Extremely satisfied	3 (1.7%)	0 (0.0%)	89 (40.3%)	81 (43.8%)		57 (47.1%)
Test-retest reliability (KW [95% CI])^*^	–	0.416 (0.253, 0.579)	0.857 (0.778, 0.937)	–		–
**Item 2 (satisfaction with intermediate vision) (range = 1 to 5)**						
Mean (SD)	2.3 (1.0)	2.2 (0.9)	3.9 (1.2)	4.0 (1.1)		4.1 (1.1)
Median	2.0	2.0	4.0	4.0		4.0
1: Extremely dissatisfied	33 (18.9%)	35 (12.9%)	11 (5.0%)	4 (2.2%)		3 (2.5%)
2: Dissatisfied	88 (50.3%)	97 (35.8%)	26 (11.8%)	22 (11.9%)		12 (9.9%)
3: Neither satisfied nor dissatisfied	26 (14.9%)	30 (11.1%)	25 (11.3%)	19 (10.3%)		10 (8.3%)
4: Satisfied	19 (10.9%)	20 (7.4%)	68 (30.8%)	57 (30.8%)		40 (33.1%)
5: Extremely satisfied	4 (2.3%)	0 (0.0%)	90 (40.7%)	83 (44.9%)		56 (46.3%)
Test-retest reliability (KW [95% CI])^*^	–	0.415 (0.251, 0.579)	0.799 (0.707, 0.891)	–		–
**Item 3 (satisfaction with distance vision) (range = 1 to 5)**						
Mean (SD)	2.2 (1.1)	2.1 (0.9)	3.9 (1.4)	4.0 (1.2)	4.0 (1.2)	
Median	2.0	2.0	4.0	4.0	4.0	
1: Extremely dissatisfied	50 (28.6%)	50 (18.5%)	20 (9.0%)	10 (5.4%)	5 (4.1%)	
2: Dissatisfied	74 (42.3%)	86 (31.7%)	26 (11.8%)	21 (11.4%)	17 (14.0%)	
3: Neither satisfied nor dissatisfied	18 (10.3%)	26 (9.6%)	20 (9.0%)	17 (9.2%)	10 (8.3%)	
4: Satisfied	24 (13.7%)	19 (7.0%)	54 (24.4%)	52 (28.1%)	29 (24.0%)	
5: Extremely satisfied	4 (2.3%)	1 (0.4%)	100 (45.2%)	85 (45.9%)	60 (49.6%)	
Test-retest reliability (KW [95% CI])^*^	–	0.391 (0.222, 0.560)	0.817 (0.730, 0.905)	–	–	
**Item 4 (satisfaction with new lens)**^**†**^** (range = 1 to 5)**						
Mean (SD)	–	–	3.8 (1.2)	3.9 (1.1)	4.0 (1.1)	
Median	–	–	4.0	4.0	4.0	
1: Extremely dissatisfied	–	–	14 (6.3%)	7 (3.8%)	4 (3.3%)	
2: Dissatisfied	–	–	22 (10.0%)	18 (9.7%)	13 (10.7%)	
3: Neither satisfied nor dissatisfied	–	–	26 (11.8%)	28 (15.1%)	9 (7.4%)	
4: Satisfied	–	–	79 (35.7%)	58 (31.4%)	43 (35.5%)	
5: Extremely satisfied	–	–	78 (35.3%)	73 (39.5%)	52 (43.0%)	
Test-retest reliability (KW [95% CI])^*^	–	–	0.879 (0.803, 0.955)	–	–	
**Item 5 (choose same lens again)**^**†**^** (range = 1 to 5)**						
Mean (SD)	–	–	4.0 (1.2)	3.9 (1.2)	3.9 (1.4)	
Median	–	–	4.0	4.0	4.0	
1: Extremely unlikely	–	–	11 (5.0%)	13 (7.0%)	12 (9.9%)	
2: Unlikely	–	–	17 (7.7%)	14 (7.6%)	13 (10.7%)	
3: Neither likely nor unlikely	–	–	34 (15.4%)	26 (14.1%)	9 (7.4%)	
4: Likely	–	–	57 (25.8%)	47 (25.4%)	32 (26.4%)	
5: Extremely likely	–	–	100 (45.2%)	83 (44.9%)	55 (45.5%)	
Test-retest reliability (KW [95% CI])^*^	–	–	0.927 (0.894, 0.960)	–		–

Note: CI: confidence interval; KW: weighted Kappa coefficient; n: sample size; SD: standard deviation.

^*^Test-retest reliability was assessed using KW and its 95% CI applied to data gathered (1) preoperatively between Visits 0A and 00 (administrations were, on average, 15.0 days apart [SD = 13.1, range = 0–88 days], *n* = 170) and (2) within Visit 3A (administrations were approximately 120 minutes apart, *n* = 209).

^†^IOL-RSS Items 4 and 5 were not administered at Visits 00 and 0A because they ask about the new lens, which was not yet implanted

**Table 5:**
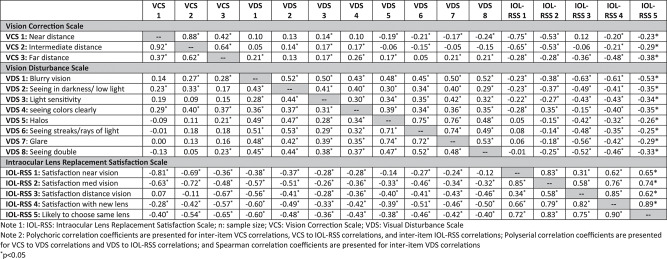
Correlations between PR-ILQ item scores at Visit 3a (above diagonal, *n* = 221) and Visit 5A (below diagonal, *n* = 121)

*Test-retest reliability*: Evaluated via the weighted Kappa coefficient (KW) for the ordinally scored items of the VCS and IOL-RSS and the intraclass correlation coefficient (ICC) for continuously scored items of the VDS, test-retest reliability assesses whether items in a questionnaire produce similar scores at different points between which no/minimal change in the respondent’s condition is expected [39]. To support use of an assessment for research purposes (i.e., the context in which the PR-ILQ was intended), guidelines suggest ICC and KW values less than 0.40 as poor, between 0.40 and 0.75 as good, and above 0.75 as excellent [40, 41], whereas others suggest ICC values less than 0.40 as poor, 0.40 to 0.59 as fair, 0.60 to 0.74 as good, and 0.75 and above as excellent [42].

As summarized in Tables 2, 3, and 4, observed results mostly indicate “fair”/“good” (i.e., KW/ICC > 0.40 to 0.60) to “excellent” (i.e., KW/ICC > 0.75) test-retest reliability for each of the VCS, VDS, and IOL-RSS items, respectively. The lower bounds of the 95% confidence intervals (CIs) around the KWs/ICCs tempers confidence in this conclusion, which is based on data gathered (1) preoperatively between Visits 0A (test) and 00 (retest, an interval that, on average, was 15.0 days apart [SD = 13.1, range = 0–88 days], *n* = 175 for the VCS and VDS and *n* = 170 for the IOL-RSS) and (2) within Visit 3A (this test-retest interval was approximately 120 minutes apart, *n* = 209). Lower pre-treatment KW and ICC values are often attributed to the lack of variability in scores (e.g., in the present study, patients entering SBL-INI-02–13 were expected to cluster at the more severe end of item response choices, resulting in a truncated range of scores that reduces reliability) [43]. However, oppositely skewed scores at Visit 3A compared to Visit 0A and 00 were still associated with much higher reliability estimates and perhaps other, non-statistical factors can be considered; for example, the difference in time interval between the two analyses (approximately 2 weeks versus 120 minutes).

*Construct-related validity*: Measurement validity is determined by theory and evidence supporting the use of assessment scores for an intended purpose [44, 45]. Here, validity conclusions were based on results from correlational methods to evaluate the extent to which PR-ILQ variables relate to other variables as expected. Correlation coefficients tabulated between PRILQ items (Table 5) and between PR-ILQ items and LogMAR scores at study visits (Table 6) were interpreted based on the following guidelines (noting that weak or strong associations can provide validity evidence depending on the nature of the relationship) [46]: |0–0.09| (negligible), |0.1–0.23| (weak), |0.24–0.36| (moderate), and | > 0.36| (strong). As a first psychometric inquiry into PR-ILQ scores, specific hypotheses regarding the magnitude of relationships were not made; however, several logic driven principles guided expectations. Broadly, it was expected that patient-reported experiences would be more strongly related to each other than to measures of VA, VCS items scores would be more strongly related to measures of VA than VDS item scores, and reports of IOL satisfaction would be more strongly related to VCS and VDS items than measures of VA. Results supported various validity hypotheses, including:

**Table 6:** Correlation between PR-ILQ item scores and visual acuity scores across timepoints

	Near visual acuity^†^		Intermediate visual acuity^†^		Far visual acuity^†^	
	3A (*n* = 221)	5A (*n* = 121)	3A (*n* = 221)	5A (*n* = 121)	3A (*n* = 221)	5A (*n* = 121)
**Vision Correction Scale**						
VCS 1: Near distance	0.76^*^	0.59^*^	0.75^*^	0.54^*^	0.80^*^	0.62^*^
VCS 2: Intermediate distance	0.64^*^	0.59^*^	0.61^*^	0.51^*^	0.62^*^	0.45^*^
VCS 3: Far distance	0.21^*^	0.23^*^	0.22^*^	0.13	0.06	0.03
**Vision Disturbance Scale**						
VDS 1: Blurry vision	-0.01	0.07	-0.06	0.00	0.11	-0.04
VDS 2: Seeing in darkness/ low light	0.14^*^	0.18^*^	0.08	0.11	0.18^*^	0.04
VDS 3: Light sensitivity	0.04	0.11	-0.01	0.14	0.04	0.04
VDS 4: seeing colors clearly	0.06	0.12	-0.09	0.11	0.03	-0.02
VDS 5: Halos	-0.25^*^	-0.01	-0.31^*^	-0.14	-0.19^*^	-0.20^*^
VDS 6: Seeing streaks/rays of light	-0.29^*^	-0.05	-0.22^*^	-0.06	-0.01	-0.02
VDS 7: Glare	-0.28^*^	0.01	-0.20^*^	-0.06	-0.06	-0.08
VDS 8: Seeing double	-0.26^*^	-0.08	-0.30^*^	-0.17^*^	-0.06	-0.14
**Intraocular Lens Replacement Satisfaction Scale**						
IOL-RSS 1: Satisfaction near vision	-0.57^*^	-0.45^*^	-0.58^*^	-0.48^*^	-0.62^*^	-0.42^*^
IOL-RSS 2: Satisfaction intermediate vision	-0.38^*^	-0.39^*^	-0.36^*^	-0.38^*^	-0.47^*^	-0.39^*^
IOL-RSS 3: Satisfaction distance vision	0.19^*^	-0.09	0.17^*^	0.02	0.07	0.11
IOL-RSS 4: Satisfaction with new lens	-0.09	-0.25^*^	-0.22^*^	-0.26^*^	-0.21^*^	-0.14
IOL-RSS 5: Likely to choose same lens	-0.18^*^	-0.30^*^	-0.20^*^	-0.27^*^	-0.29^*^	-0.14

Note 1: IOL-RSS: Intraocular Lense Replacement Satisfaction Scale; n: sample size; VCS: Vision Correction Scale; VDS: Visual Disturbance Scale. Note 2: Polyserial correlation coefficients are presented for VCS and IOL-RSS correlations; Spearman correlation coefficients are presented for VDS correlations. *p<0.05

^†^Uncorrected for both eyes (note, for statistical analysis, ETDRS scores were converted using a LogMAR scale, which calculates visual acuity based on the total number of letters read incorrectly corresponding to the last line from which the subject read at least one letter correctly, i.e., the baseline LogMAR visual acuity). A LogMAR value represents the amount of vision loss with a score of zero indicating no vision loss, higher scores indicative of poorer vision, and negative scores indicative of better vision. Unique and independent LogMAR scores were generated for uncorrected near, intermediate, and distance visual acuity

- **Relationships between use of vision correction and satisfaction with vision (Table**5): It was expected that individuals reporting less reliance on the use of vision correction at near, intermediate, and far distances would be more satisfied with their ability to see things at those respective distances. This hypothesis was supported based on data collected at Visits 3A and 5A, which show increasingly strong relationships between reliance on vision correction at near (VCS-1/IOL-RSS-1, *r* = −0.75, −0.81), intermediate (VCS-2/IOL-RSS-2, *r* = −0.53, −0.72), and far (VCS3/IOL-RSS-3, *r* = −0.36, 0.67) distances and satisfaction with ability to see at these distances, respectively. Importantly, correlation coefficients between related variables on the VCS and IOL-RSS were more strongly related to each other than adjacent variables. For example, VCS-1 was more strongly related to IOL-RSS-1 (*r* = −0.81) than to IOL-RSS-2 (*r* = −0.63) and IOL-RSS-3 (*r* = 0.07), VCS-2 was more strongly related to IOL-RSS-2 (*r* = −0.72) than IOL-RSS-1 (*r* = −0.69) and IOL-RSS-3 (*r* = −0.11), and VCS3 was more strongly related to IOL-RSS-3 (*r* = −0.67) than IOL-RSS-1 (*r* = −0.36) and IOL-RSS-2 (*r* = −0.48).
- **Relationships between use of vision correction and visual acuity (Table**6): At Visits 3A and 5A, strong relationships were observed between reliance on vision correction at near (VCS-1/VA-near, *r* = 0.76, 0.59) and intermediate (VCS-2, VA-intermediate, *r* = 0.61, 0.51) distances and LogMAR scores at these distances, respectively. Negligible relationships, however, were observed between reliance on vision correction at far distances (VCS-3) and LogMAR scores at far distances (*r* = 0.06, 0.03).
- **Predictors of overall lens replacement satisfaction (Tables**5 and 6): Increasingly strong relationships between lens satisfaction (IOL-RSS-4) and reduced use of vision correction at near (VCS-1, *r* = −0.20, −0.28), intermediate (VCS-2, *r* = −0.21, −0.42), and far (VCS-3, *r* = −0.48, −0.57) distances were observed at Visits 3A and 5A. Additionally, reduced visual disturbances strongly related to overall lens satisfaction (IOL-RSS-4), with reduced blurriness (VDS-1, *r* = −0.61, 0.60), streaks or rays of light (VDS-6, *r* = −0.35, 0.51), and double vision (VDS-8, *r* = −0.46, 0.50) showing among the strongest relationships (Table 5). LogMAR scores (i.e., estimates of VA) tended to be only moderately related to overall lens satisfaction (IOL-RS-4) at near (*r* = −0.09, −0.25), intermediate (*r* = 0.022, −0.26), and far (*r* = −0.21, 0.14) distances at both Visits 3A and 5A.

*Sensitivity to change*: Sensitivity-to-change reflects the extent to which scores from a questionnaire fluctuate with actual change in the concept it represents. In this analysis, sensitivity to change was assessed correlationally by relating PR-ILQ item change scores to each other and with indicators of VA (i.e., LogMAR scores). Hypotheses in this regard were based on several principles as described just above (e.g., patient reports would be related more strongly with each other than with indicators of VA, VCS item and change scores would be more strongly related to change in VA than VDS item scores). As anticipated, change scores between respective VCS and IOL-RSS items were moderately to strongly correlated (Table 7). Changes at Visit 5 from Baseline in VCS-1 (near), VCS-2 (intermediate), and VCS-3 (far) distances correlated as expected to changes in satisfaction with the ability to see things at near (IOL-RSS-1, *r* = −0.53), intermediate (IOL-RSS-2, *r* = −0.42), and far (IOL-RSS-3, *r* = −0.40) distances, respectively.

At baseline, only VA for uncorrected left and right eye separately at far distance was evaluated; therefore, it was not possible to directly test many PR-ILQ sensitivity-to-change hypotheses in terms of its relationship with change in VA. Nevertheless, several supportive results emerged. First, change at visit 5A from baseline in VCS-3 (far distance) had moderate relationships to change in VA at far distance (*r* = 0.28 and 0.24 with left and right eye, respectively) whereas change in VCS-1 (near) and VCS-2 (intermediate) scores had negligible relationships with change in far distance VA for both the left (*r* = 0.07, 0.13) and right (*r* = −0.01, 0.09) eyes. Similarly, and also as anticipated, change at Visit 5A from Baseline in IOL-RSS-3 (satisfaction seeing far distance) had strong relationships with change in far distance VA for both the left (*r* = −0.44) and right (*r* = −0.38) eyes, whereas IOL-RSS-1 (near) and IOL-RSS-2 (intermediate) scores were less strongly related with change in far distance VA for both the left (*r* = −0.22, −0.25) and right (*r* = 0.09, −0.33) eyes, respectively. Note: VA is based on LogMAR values representing the amount of vision loss, with a score of zero indicating no vision loss, higher scores indicative of poorer vision, and negative scores indicative of better vision.

**Table 7:**
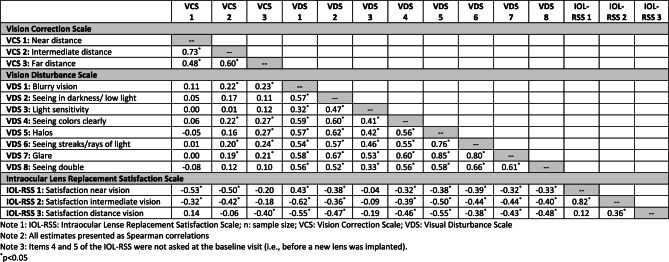
Correlation coefficients among PR-ILQ change scores at Visit 5A from Baseline (*n* = 121)

## Discussion

The demand for PRO questionnaires in ophthalmic research and practice continues to grow. In response, the PR-ILQ was created explicitly for use in IOL replacement clinical research to evaluate outcomes among adults with cataracts seeking IOL replacement. The PR-ILQ was developed in ways consistent with regulatory guidance [28, 29] and best measurement practices [30, 31], with input from ophthalmology experts (*N* = 10) and over 70 patients with cataracts (pre- and post-IOL implantation patients). These activities support the content validity of the PR-ILQ, indicating it assesses concepts relevant to cataracts and that are important to patients undergoing IOL replacement treatment, and that patients can provide responses reflecting their eye health and functional status, visual disturbances, and satisfaction with the implanted lens. Upon establishing its content validity, the PR-ILQ was implemented in a clinical study (SBL-INI-02–13) of patients with bilateral cataracts undergoing IOL replacement (*N* = 271). As a first inquiry into the psychometric performance of the PR-ILQ, analyses demonstrated that respondents endorsed the range of item responses and provides early evidence of reliability and validity of its scores.

The patient-centric, measurement-focused, and theoretical value of using the PR-ILQ as an outcome assessment in IOL replacement trials can be supported by the content validity and psychometric evidence presented herein. Together this gives researchers confidence the PR-ILQ can have real-world and practical benefits. The real-world and practical benefits were then realized when this evidence was considered by device developers, IOL researchers, and regulatory reviewers both prior to and during the review of results from the pivotal SBL-INI-02–13 trial designed to evaluate the safety and efficacy of the SBL-3™ Multifocal Posterior Chamber Intraocular Lens (MIOL) intended for adults with cataracts.

As detailed in the SBL-3™ MIOL regulatory-approved physician labeling guide and elsewhere [21, 27], the PR-ILQ was used in in several ways in SBL-INI-02–13. First, the VCS items supported PRO-based secondary effectiveness claims. Specifically, more patients in the SBL-3™ group reported reduced use of vision correction versus the comparator to help see things at near (93.3% versus 45.5%), intermediate (93.9% versus 45.3%), and far (93.9% versus 85.1%) distances. A treatment responder was defined as those who reported either “never used vision correction” or “used vision correction some of the time” on VCS-1 (near), VCS-2 (intermediate), and VCS-3 (far).

In addition to reflecting the IOL’s benefit to the patient, the patient-centric language of the VCS helped evolve previous IOL physician labeling materials. These materials often stated the IOL was indicated for “patients who *desire* [emphasis added] near, intermediate, and distance vision with increased spectacle independence” [7](p.1)]. Newer language indicates that “the lens promotes the less frequent use of vision correction choices at near distance (including glasses, contact lenses, magnifying glasses, and digital adjustments on electronic devices)” [21].

Secondly, IOL-RSS items assessing satisfaction with ability to see things at near, intermediate, and far distance informed secondary endpoints in the SBL-3™ development program. These results are summarized in the FDA-approved physician label; however, label claim language is only associated with use of vision correction options [21]. Specifically, more patients in the SBL-3™ group reported satisfaction versus the comparator in terms of their ability to see at near (89.2% versus 47.2%) and intermediate (89.2% versus 66.5%) distances, but less satisfaction at far (76.4% versus 90.7%) distance. A treatment responder was defined as those who reported either being “satisfied” or “extremely satisfied” on IOL-RSS Item 1 (near), 2 (intermediate), and 3 (far), respectively.

Thirdly, results from the VDS as administered in SBL-INI-02–13 were summarized in the Physician Labeling section, Other Safety Endpoint Outcomes. VDS items assess specific visual disturbances (e.g., blurry vision) on a 0 to 10 NRS, and item-level scores were summarized descriptively to allow visual inspection of between-group mean differences within study visits and within-group changes across study visits. For example, the VDS Item 1 (blurry vision) mean scores for the SBL-3™ and comparator groups at Baseline, Visit 4a, and Visit 5a were 6.27 and 6.44, 2.83 and 2.16, and 2.43 and 2.43, respectively, indicating similar reductions post-implant. In anticipation of more recent regulatory guidance [47], VDS item scores were transformed to a VRS to create meaningful score regions and show, perhaps in a simpler or more intuitive fashion, the number of subjects within each group reporting their visual disturbances as “None” (NRS = 0), “Mild” (NRS = 1 to 3), “Moderate” (NRS = 4 to 6), and “Severe” (NRS > 6). This scaling helped support interpretations regarding the meaning of patient-reported visual disturbance changes over time.

In terms of strengths, PR-ILQ content was developed with input from over 70 patients with cataracts (pre- and post-IOL implantation), ensuring it measures experiences relevant to disease and important to the patients who are receiving lens replacement. The psychometric evaluation of the PR-ILQ was based on a large sample of patients in a randomized, well-controlled clinical trial, meaning results are based on data collected at multiple timepoints and in the context of use for which the assessment was designed. Finally, that the substantive PR-ILQ results generated from the clinical trial contributed to a new product approval decision, secondary effectiveness claims, and safety observations indicates that the FDA considers the assessment to have value and that the patient voice matters when developing new treatments.

The PR-ILQ was developed to evaluate the experiences of patients pre- and post-implantation of a segmented bifocal lens, specifically an asymmetric segmented multifocal lens (sometimes called extended depth of focus lens). Though there is some confusion in categorization [48], other types of IOL replacements exist (e.g., monofocal and toric IOLs). In anticipation of its potential use in other lens type evaluation studies, patients interviewed as part of the PR-ILQ development had various lens types. Nevertheless, the assessment may be limited in its ability to assess concepts important to patients with other IOL types.

The PR-ILQ may also be limited in terms of its utility for younger patients. That is, the research contributing to PR-ILQ development and evaluation was with adults ≥18 years old, typically later in life. As IOL options become increasingly available for pediatric cataracts, researchers should consider its appropriateness for younger patients. Additionally, future researchers may wish to use the PR-ILQ in other contexts (e.g., non-English languages and/or clinical settings) and/or using alternative modes of administration (e.g., electronic); therefore, potential barriers for use of the assessment in these other contexts would need to be explored, and translation and linguistic validation activities [49] would need to be considered.

Reliability is a property of scores (and not questionnaires, per se) [50], and validity characterizes the confidence researchers can have in the meaning and inferences ascribed to those specific scores [45, 50, 51]. Therefore, sets of interrelated psychometric-focused inquiries are always necessary to understand reliability and validity of PRO measures, particularly a newly developed one [35]. Though the present results provide early support for the conclusion that the PR-ILQ can produce reliable scores in the target population and that researchers can have confidence in the validity of inferences drawn from those scores, researchers are also encouraged to report its psychometric characteristics in future inquiries. Such inquiries may include exploring reliability concerns raised in the present inquiry (e.g., lower pre-treatment reliability on the IOL-RSS Items 1, 2, and 3) as well as ubiquitous measurement considerations not addressed herein. For example, future research on the PR-ILQ could explore how confounders (e.g., age, type of IOL), missingness, or response bias (i.e., systematic errors in responses not directly related to health status concepts of measurement) may impact psychometric performance and the generalizability of results to real world patients undergoing IOL surgery; item independence; and creation of score interpretation guidelines (e.g., via distribution- and anchor-based methods). Lastly, it would be of value to explore how use of alternate scoring methods may enhance the assessment’s utility outside of clinical trials (e.g., researchers may wish to consider creating a VCS, VDS, or IOL-RSS scoring system beyond the current item level scoring as presented herein).

## Conclusion

The PR-ILQ was developed in the context of previous research indicating that PRO questionnaires are underutilized in new medical device evaluations including for new IOLs [22]. Results presented here support the assessment’s content validity (i.e., it measures concepts relevant to cataracts and important to patients undergoing IOL replacement treatment and can do so in ways that respondents easily understand) and psychometric performance (i.e., it produces reliable scores when administered in the target patient population, and inferences drawn from those scores can trusted as valid), though additional research is warranted. That the assessment has recently been used to support a new product approval decision, secondary effectiveness claims, and safety observations indicates that the PR-ILQ, along with the evidentiary basis presented herein, will be of immediate value to IOL replacement outcomes researchers, regulators, and other stakeholders interested in generating evidence to inform health care decisions and, ultimately, improve patients’ lives.

## Data Availability

The data that supports the findings of this study are available, with some restrictions, upon reasonable request with permission from Lenstec, Inc. Requests can be made to the last author.

## Abbreviations

CD: cognitive debriefing
CE: concept elicitation
CGIRB: Copernicus Group Independent Review Board®
CI: confidence interval
COA: clinical outcome assessment
ETDRS: Early Treatment Diabetic Retinopathy Study
FDA: Food and Drug Administration
ICC: intraclass correlation coefficient
IFU: indication for use
IOL: intraocular lens
IOL-RSS: IOL Replacement Satisfaction Scale
NRS: numeric rating scale
PR-ILQ: Patient-Reported Intraocular Lens Questionnaire
PRO: Patient-reported outcome
SD: standard deviation
UK: United Kingdom
US: United States
VCS: Vision Correction Scale
VDS: Vision Disturbance Scale
VRS: verbal rating scale
KW: weighted Kappa coefficient
